# Late Recovery from Severe* Streptococcus pneumoniae* Comatose Meningitis with Concomitant Diffuse Subcortical Cytotoxic Edema and Cortical Hypometabolism

**DOI:** 10.1155/2018/9439021

**Published:** 2018-10-09

**Authors:** Philippe Hantson, Thierry Duprez

**Affiliations:** ^1^Department of Intensive Care, Cliniques St-Luc, Université Catholique de Louvain, Brussels, Belgium; ^2^Department of Neuroradiology, Cliniques St-Luc, Université Catholique de Louvain, Brussels, Belgium

## Abstract

A 75-year-old woman was admitted to ICU with coma following* Streptococcus pneumoniae *meningitis with bacteremia. Her Glasgow Coma Scale (GCS) score fluctuated around 4 to 6 over the next four weeks. There was no evidence of increased intracranial pressure (ICP). Electroencephalogram (EEG) showed only diffuse aspecific slowing. Impaired cerebral blood flow (CBF) autoregulation was suggested at transcranial Doppler (TCD). Repeated brain magnetic resonance imaging (MRI) examination failed to demonstrate venous thrombosis, arterial ischemic stroke, or brain abscesses but revealed diffuse but reversible cortical cytotoxic edema at diffusion-weighted (DW) sequences. The brain FDG-positron emission tomography (FDG-PET) showed diffuse cortical hypometabolism. The patient unexpectedly experienced a complete neuropsychological recovery the next few weeks. The suggested hypothesis to explain this unusual disease course could be a transient alteration of CBF autoregulation due to some degree of diffuse subcortical microangiopathy. A concomitant reduction of brain metabolism probably prevented the progression towards cortical irreversible ischemic damage.

## 1. Introduction

*Streptococcus pneumoniae* meningitis still remains a severe condition leading to a high mortality and morbidity rate in spite of optimal intensive care management. Poor issue usually results from delayed ischemic damage due to inflammation and (micro)vascular thrombosis [1–8]. But a few patients experience a good neurological outcome despite an initially severe and even prolonged coma. A careful analysis of such cases could allow insights into the pathophysiology of brain perfusion and metabolic response at the acute phase in patients experiencing recovery.

## 2. Case Report

A 75-year-old woman (weight: 72 kg) was admitted to the Emergency Department for agitation with an altered consciousness. Patient's medical history was unremarkable, except for arterial hypertension treated by atenolol. Symptoms started acutely a few hours earlier, with a progressive loss of verbal contact. On admission, the Glasgow Coma Score (GCS) score was 9/15 (E4, V1, M4), with moderate neck stiffness. There was no lateralized deficit and pupils were mid-size, reactive, and symmetric. Vital signs were as follows: body temperature of 36.6°C, arterial blood pressure of 180/95 mm Hg, heart rate of 120/min, and respiratory rate of 60/min. A brain computed tomography (CT) without iodinated contrast agent (CA) perfusion before lumbar puncture did not reveal any significant abnormality. Intubation was required because of progressive respiratory distress. The cerebrospinal fluid (CSF) analysis revealed white blood cells (WBC) count at 560/*μ*l, with 99% granulocytes, proteins at 1264 mg/dl, glucose at 3 mg/dl, and lactate at 27 mmol/l.

The CSF and blood cultures grew positive for* Streptococcus pneumoniae* sensitive to penicillin G and ceftriaxone. The minimal inhibitory concentration (MIC) was 0.016 mcg/ml for penicillin G and ceftriaxone in the CSF and 0.012 and 0.008 mcg/ml in blood for penicillin G and ceftriaxone, respectively. Treatment combining dexamethasone (10 mg q6h for 4 days) and ceftriaxone (2 g q12h for 14 days) was initiated. CSF analysis was repeated after 10 days and confirmed both a drop in WBC count and eradication of the causative microorganism.

**Figure 1:**
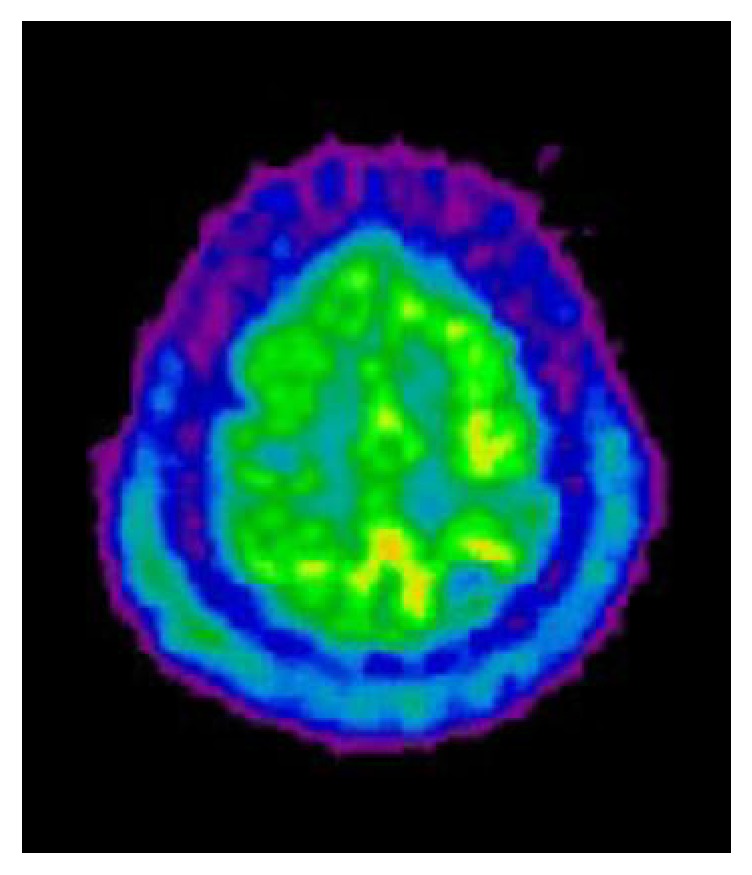
Brain ^18^fluorodeoxyglucose (FDG) positron emission tomography (PET) showing diffuse cortical hypometabolism featured by decreased uptake of the tracer.

The patient was subsequently referred to the Intensive Care Unit (ICU) because of worsening of GCS score at 6/15 (E1, V1, M4). Despite the lack of evidence of acute hydrocephalus, intracranial pressure (ICP) was monitored by intraventricular catheter and remained within the normal range during the whole ICU stay. The mean arterial blood pressure was around 80 mm Hg. No sedative drugs were required for mechanical ventilation and GCS score remained stable at 6/15. A control brain CA-enhanced CT after 72 hours of therapy failed to reveal brain abscesses, thrombosis, or ischemic lesions. The patient was repeatedly examined by electroencephalogram (EEG) in order to exclude nonconvulsive status epilepticus. There was only diffuse slowing with predominance of delta and theta waves, together with some triphasic activity. A brain positron emission tomography (PET) using ^18^fluorodeoxyglucose (FDG) as tracer was performed on day 13 and was consistent with a diffuse cortical hypometabolism and relatively preserved uptake within grey nuclei (Figure 1). A transcranial Doppler (TCD) examination performed at day 3 suggested that cerebral autoregulation at different levels of mean arterial pressure was abolished. There was no increase in cerebral blood flow velocity (CBFV).

Brain magnetic resonance imaging (MRI) was performed on days 8, 11, and 30. On day 8, while the diffusion-weighted imaging (DWI) and T2/fluid attenuated inversion recovery (FLAIR) sequences were not significantly modified (not shown), there was a marked decrease (300-500 instead of 700.10^−6^ mm^2^.sec^−1^) in the apparent diffusion coefficient (ADC) diffusely in the subcortical areas, at both the supratentorial and infratentorial levels. This finding was suggestive of cytotoxic edema of U-fibers and immediately adjacent superficial white matter. The picture was relatively unchanged on day 11; magnetic resonance spectroscopy in the areas with low ADC values failed to retrieve any peak of lactate. On day 30, while the patient was still comatose, ADC values in the subcortical territories had returned to normal range and no ischemic damage within overlying cortex had appeared.

The patient remained in deep coma (GCS from 4 to 6/15) for more than four weeks but then started progressively to wake up, with eye opening, and became able to understand verbal command.

She presented two episodes of pneumonia during the ICU stay: a first episode with methicillin-resistant* Staphylococcus aureus* (present at admission screening in the nose and throat sampling) and thereafter a relapse* with Pseudomonas aeruginosa* which had also been initially detected in the throat. Blood cultures remained negative and the patient did not develop septic shock or acute renal failure. However, due to the extension of nosocomial pneumonia, it was necessary to ventilate the patient with 0.5 FiO_2_ for a long period. Hypoxemia was never observed. Neuromuscular blocking agents were not used during mechanical ventilation. Thus far, in our opinion, the delayed neurological recovery was independent from these infectious complications. The patient stayed in the ICU for a total of 64 days, mainly because of a difficult weaning from the ventilator due to nosocomial pneumonia and critical illness polyneuropathy. At 6-month follow-up, the neuropsychological testing confirmed excellent recovery.

## 3. Discussion

Cerebrovascular complications are well described during the course of* S. pneumoniae *meningitis [1]. They may extend from cerebral infarction within the first days of the disease to delayed cerebral ischemia that may even occur in patients with an apparently good initial recovery [2–8]. Some reports indicate that vascular complications may occur later on, even months after the meningitis, due to the development of intracranial vascular stenosis [6].

Investigations by TCD in patients with acute bacterial meningitis have shown that patients with lower GCS score at admission had a higher incidence of elevated CBFV (>150 cm/sec) and a higher incidence of ischemic stroke [9]. Arterial vascular narrowing was documented in a significant number of patients, but not all, suggesting that other vascular alterations could occur. The ischemic complications are likely related to the development of a cerebral vasculopathy triggered by the presence of purulent material. In addition to arterial brain vessel narrowing that can be seen at angiography, there is also evidence of widespread microangiopathy involving the primary inflammatory reaction, immune mechanisms, and microthrombosis [10]. The benefit of corticosteroids therapy in* S. pneumoniae *meningitis must not be underestimated [11]. However, it seems difficult to demonstrate that the protective effect of dexamethasone administration is linked to a reduction of ischemic complications [12, 13].

The spectrum of pathological brain MRI was investigated in 136 patients with documented purulent bacterial meningitis (*S. pneumoniae *in 52.6%) [14]. The involvement of cortical parenchyma (20%) and white matter (26.7%) was found in a substantial amount of cases. DWI appeared as a sensitive technique to detect cortical and/or white matter cytotoxic edema. Subcortical low intensity on T2/FLAIR MR images has also been highlighted in a minority of cases of bacterial meningitis [15]. This feature is present at the acute stage and is usually transient. The mechanism beyond this transient subcortical low intensity appears unclear in meningeal diseases. A paramagnetic substance as seen in free radicals could be involved as oxygen free radicals have been implicated as pathologic mediators in experimental models of pneumococcal meningitis [16]. The subcortical low-intensity lesions may have intermediate to low signal intensity on DW images, with the measured ADCs slightly lower than those on the normal white matter. The reason for low ADCs despite poorly modified DW images could be consistent with the accumulation of free radicals in the subcortical white matter rather than with structural lesions [15].

In the present case, central nervous system dysfunction could be related not only to meningitis but also to sepsis [17]. However, the depth and duration of coma were unexpectedly long for sepsis-related encephalopathy. The EEG findings could be consistent with this hypothesis, but they remain unspecific. Regarding MRI, patients with septic shock who develop delirium, coma, seizures, or a focal deficit exhibit evidence of white matter hyperintensities (21%) or ischemic stroke (18%) [18]. White matter hyperintensities may be associated with long-term cognitive impairment. Ischemic stroke is associated with coma, focal neurologic signs, and unfavorable outcomes. Conflicting studies have addressed the influence of sepsis on cerebral blood flow (CBF), and experimental studies have highlighted either increased or decreased CBF. In rats, pneumococcal bacteremia triggered cerebral vasodilation but did not entirely abolish CBF autoregulation in the absence of meningitis [19]. In contrast, CBF autoregulation was lost after intracisternal inoculation of the agent and subsequent meningitis [20]. As our patient suffered systemic hypertension, the theoretical risk for critical hypoperfusion and ischemia was higher during the episodes of low blood pressure. The management of arterial blood pressure was therefore adapted.

## 4. Conclusion

Despite deep initial and sustained coma, good functional recovery remains possible in some patients suffering from severe* S. pneumoniae *meningitis. In this setting, prolonged coma is not related to the development of diffuse brain abscesses, ischemic damage, vascular thrombosis, or hydrocephalus with increased ICP. Combined brain imaging using MRI and PDG PET could be helpful to assess concomitantly some degree of diffuse but reversible cytotoxic edema (MR diffusion-weighted imaging), more probably due to hypoperfusion together with life-saving diffuse cerebral hypometabolism (FDG-PET) preventing the development of irreversible ischemic damage. This could be considered as a form of reversible microangiopathy, but it remains impossible to affirm that dexamethasone could be helpful in the condition.
